# Considerations for Neural Cannabis Cue-Reactivity Studies in Individuals With Cannabis Use Disorder

**DOI:** 10.1016/j.bpsgos.2025.100669

**Published:** 2026-01-12

**Authors:** Erica N. Grodin, Kaitlin McManus

**Affiliations:** a Department of Psychiatry and Biobehavioral Sciences, University of California Los Angeles, Los Angeles, California; b Brain Research Institute, University of California Los Angeles, Los Angeles, California; c Department of Psychology, University of California Los Angeles, Los Angeles, California

The prevalence of cannabis use disorder (CUD) is rising; however, a mechanistic understanding of continued cannabis use in individuals with CUD is lacking. In their recent study published in *Biological Psychiatry: Global Open Science*, Lorenzetti *et al.* (1) sought to elucidate the neurobiology of cannabis cue-reactivity and craving in individuals with moderate-to-severe CUD and past cannabis reduction/quit attempts. The authors examined brain activity during a neural cannabis cue-reactivity task in individuals with CUD compared with control individuals who did not use cannabis. They extended this work by exploring associations between cannabis cue–induced brain activity and clinical symptomatology of CUD including subjective cannabis craving, self-reported arousal, cannabis withdrawal symptoms, and CUD symptom severity, as well as with biomarkers of recent cannabis use. Lorenzetti *et al.* (1) found that compared with control individuals, individuals with CUD had greater neural activity when viewing cannabis cues versus neutral images in regions of the brain responsible for salience/reward, attentional processing, and inhibitory functioning (i.e., occipital, orbitofrontal, cingulate, cerebellar, hippocampal, middle temporal, lateral parietal cortices). Furthermore, clinical symptomatology including self-reported arousal and withdrawal symptoms was positively correlated with occipital and cerebellar activity, whereas Δ^9^-tetrahydrocannabinol levels were negatively correlated with activity in the anterior cingulate and inferior parietal cortices. Together, Lorenzetti *et al.* (1) provide critical insights into the mechanistic understanding of cannabis craving in individuals with moderate-to-severe CUD who have attempted to reduce/quit cannabis use, including observations that 1) neural activity in response to cannabis cues in individuals with CUD is overlapping with the neurobiology of craving in other substance use disorders and that 2) interventions targeting executive and reward functioning may be effective for those with CUD who are vulnerable to relapse (1).

### Considerations for Conceptualizing Subjective and Neural Cannabis Craving

Lorenzetti *et al.* (1) found that subjective craving for cannabis increased from pre- to postneural cannabis cue-reactivity task, but subjective cannabis craving was not correlated with cannabis cue–induced brain activity. The authors posit that the absence of a correlation may be due to factors modulating subjective craving such as perceived availability of cannabis, CUD severity, or low variability in subjective craving self-reports (1). It is not uncommon for neuroimaging cue-reactivity studies to observe null associations between subjective craving and neural cue–induced brain activation. Although neural cue-reactivity has traditionally been theorized to represent a biological indicator of craving, it has been posited that neural cue-reactivity tasks may not induce clinical levels of craving but rather capture biological reactions to conditioned substance stimuli (2,3), as brain regions involved in learning and memory, visual perception, and salience and attentional processing are commonly activated by drug cues. In the study by Lorenzetti *et al.* (1), methodological considerations may have further inhibited the ability to observe significant associations between behavior and biology, as cannabis craving was assessed immediately before and after completion of the functional magnetic resonance imaging (fMRI) task (1). However, it has been noted that perceptions of the psychological craving state change rapidly (4), and therefore, fMRI substance cue-reactivity guidelines suggest implementing in vivo assessments of craving during the cue-reactivity task while individuals are in the scanner (5). In-scanner cannabis craving assessments may be especially useful when considering the concerns related to whether neural cue-reactivity studies elicit clinical levels of craving (2,3). Therefore, future research in this area should consider utilizing in vivo subjective craving assessments during fMRI cue-reactivity tasks. An additional methodological consideration includes the types of stimuli used during substance cue-reactivity tasks. It may be posited that the neurobiological response to cannabis cues observed by Lorenzetti *et al.* (1) is indicative of general reward and salience activation rather than cannabis-specific craving. Therefore, future research should consider the inclusion of rewarding non–substance comparator images (e.g., monetary images) to identify pathological levels of neural activation for cannabis cues versus non–cannabis cues. Doing so would elucidate whether neural activation patterns during cue-reactivity tasks in individuals with CUD are related to cannabis specifically or to reward more generally, which is currently an open question in the field.

Furthermore, the psychometrics of the cannabis craving assessment should be considered. The authors assessed cannabis craving with a single-item visual analogue scale asking individuals to rate “how much do you feel like smoking cannabis right now?” on a scale of 1 to 10 (1). The use of the term “smoking” in the assessment of cannabis craving is an important consideration for future research. Modes of cannabis use include not only smoking cannabis but also ingesting products containing cannabis (i.e., edibles, drinks, tinctures) and using a vaporizer or e-cigarette containing cannabis, which have become increasingly popular (6). Consideration should be given to the preferred mode of use of cannabis for individuals with CUD, and future work may consider assessing cannabis craving holistically rather than focusing on smoking cannabis. This area may also be of interest for the selection of cannabis cues for the neural cue-reactivity paradigm, which were also focused on the smoking mode of administration. It may be important to include cues that are specific to an individual’s preferred mode of administration to induce the greatest neural activation and subjective craving response. In addition to considerations around mode of use, there is a recommendation to utilize specific “craving” terminology (5,7). Therefore, a consideration may be made to assess cannabis craving by asking individuals to rate “how much do you crave using cannabis right now?,” although psychometric validation of such a cannabis craving assessment is required. Therefore, methodological consideration should be given to the way future researchers assess cannabis craving.

### Importance of the Visual Attention System in Neural Cue-Reactivity

Participants with CUD had greater cannabis cue-elicited activation in multiple brain regions, including visual and attentional areas (1). Intriguingly, activation of the superior occipital cortex, but not reward regions, was positively correlated with arousal ratings for cannabis minus neutral cues and cannabis withdrawal scores (1), indicating that greater visual cortex activation was associated with greater excitement for cannabis cues and greater withdrawal symptoms. These findings highlight the importance of higher-order visual processing in visual substance cue-reactivity fMRI research. Increased activation in response to substance-related cues in brain regions responsible for visual processing is commonly reported in studies of people with CUD (8) and other substance use disorders; however, the importance of these activation patterns and their relationship to cannabis use behaviors are rarely discussed. The association between visual processing activation and arousal (i.e., excitement) to cannabis cues may indicate that individuals with CUD are attributing greater attentional bias, due to increased incentive salience, toward visual cannabis cues. Attentional bias can be measured through visual gaze tracking during an fMRI scan and the calculation of fixation time. To assess whether greater cannabis cue-elicited activation of higher-order visual processing regions is clinically relevant, attentional bias indices could be easily incorporated into a cannabis cue-reactivity fMRI study. As attentional bias toward drug cues has been suggested as a treatment target (9), this may be an important next step in cannabis cue-reactivity research.

### Conclusions

Lorenzetti *et al.* have made substantial contributions to the understanding of neural cannabis cue-reactivity in individuals with CUD by highlighting the overlapping neurobiology between cue-induced brain activation in individuals with CUD compared with individuals with other substance use disorders and through the identification of brain-behavior relationships. There are several future directions related to the methodological assessment of craving, both subjectively and neurobiologically, including the inclusion of in vivo craving assessments, the inclusion of preferred mode of cannabis use, and the inclusion of rewarding non–cannabis control images, which may further our understanding of the neurobiology of cannabis craving in this sample. Finally, the consistent activation of visual attentional regions during substance cue-reactivity fMRI paradigms implicates attentional bias processes as being important in cue-reactivity processing. Ultimately, this work suggests that interventions targeting attention and salience circuitry in addition to typical reward circuitry may be most effective at reducing cannabis craving and use.
